# Supplementary material for: An Efficient CRISPR/Cas9 Genome Editing System for a Ganoderma lucidum Cultivated Strain by Ribonucleoprotein Method
Source: J Fungi (Basel). 2023 Dec 5;9(12):1170. doi: 10.3390/jof9121170 (PMC10745038; doi:10.3390/jof9121170)
Supplement: Supplementary file 1 [file jof-09-01170-s001.zip › jof-2722425-supplementary.pdf]

# Supplementary Materials

**Supplementary Data S1:** The sgRNA transcription cassettes (T7 promoter-Spacer-sgRNA scaffold) of *ura3*, *cyp512a3* and *cyp5359n1*.

**> *ura3***

TTCTAATACGACTCACTATAGGCCTCTTCCGTGTATGAGCGTTTTAGAGCTAGAAATAGC
AAGTTAAAATAAGGCTAGTCCGTTATCAACTTGAAAAAGTGGCACCGAGTCGGTGCTT
TT

**> *cyp512a3***

TTCTAATACGACTCACTATACGGACGGCCCACCAACTGTGGTTTTAGAGCTAGAAATA
GCAAGTTAAAATAAGGCTAGTCCGTTATCAACTTGAAAAAGTGGCACCGAGTCGGTGC
TTTT

**> *cyp5359n1***

TTCTAATACGACTCACTATAGGGTTTGCGCTTCATAGTATGTTTTAGAGCTAGAAATAGC
AAGTTAAAATAAGGCTAGTCCGTTATCAACTTGAAAAAGTGGCACCGAGTCGGTGCTT
TT

**Supplementary Data S2:** Sequences amplified from the L1 genome using primers *ura3*-F/*ura3*-R, *cyp512a3*-F/*cyp512a3*-R, or *cyp5359n1*-F/*cyp5359n1*-R. The underline indicated the full length of the gene, and the exons were marked with red letters. The PAM sequence and sgRNA-guiding sequence were highlighted in yellow and green, respectively. The red triangles indicated the cleavage site.

**> Sequence amplified using primers *ura3*-F/*ura3*-R.**

The sequence was 1074 bp long and contained the *ura3* gene full-length of 947 bp, which consisted of three exons.

AATCTCACAAGTCACCCAGCAACCACCCGTCGGCCACAAAACAAAATTGAAAGAATG
GTGGCCGTGGCCAAGCAAACATACGCGCAGAGGGCCACCAGACATCCCAACCCAGCC
GCGAAAGCTCTCCTCGAGACGATCGAGCGCAAGCGCACAAATCTGTCTGTTAGCGTCG
ATGTGACGAAACGGGAGGACTTCTTCAGGATTGTGGACATCGTGGGTCCATACGTCTG
CCTAGTAAAGGTAGGTGTACTATGTCCAAATGGTTGATATGCACTTGGGTGAATATAATC
CAAAGACCCACATAGACATCATCGAGGACTTTGATCCATCCGCGATCGAACGCCTCAA
GGCACTCAGCGAGAAGCATGACTTCCTCATCTTTGAAGACAGAAAATTTGCCGACATT
GGTAGGATGTAGTCCCTTTGCGCCCGCTACATACGATGTTGACAAGAATATAGGAAACA
CGGTGGCACTTCAATATTCTGCAGGCGTGCACAAAATCGCTAGCTGGTCGCACATCAC
GAACGCACACCCTGTCCCAGGACCGTCCATCATCTCCGGCCTCAAGGCTGTCGGTCTG
CCCCTTGGCAGGGGGCTTCTGCTCCTCGCCGAGATGAGCACCAAGGGCAGCCTCGCA
ACCGGCTCATACACGGAAGAGGCCGTCCGTATGGCTCGCGCCAACCGCGACTTCGTGA
TCGGCTTCATTGCCCAGCAACGCATGGACAGCGTCGGTCTGCGGGAGGGCGAGTCCTC
GCCGGACGAAGACTTCCTTATCCTTACTCCAGGAGTCGGACTGGATACTAGGGGAGAT
AGCATGGGGCAGCAATACCGAACGCCAAGGGAGGTGGTCATCGAGTCCAATTGCGATG
TCATCATAGTTGGTCGGGGTGTTTACGGGAACGATAACGGCACGAATGCGGAGGCAGT
CCGCGCACAGGCGGAGAGGTACCGCGCAGAAGGCTGGAAGGCATACCAGGAAAGGG
TTGGGATCTCGGATTAGGGCTAGCATTGTGCAGAGACGGGTATAGTAGCACAGTGTATA
TGTAAACAAAACATCCTACCGCTACAATCGC

**> Sequence amplified using primers *cyp512a3*-F/*cyp512a3*-R.**

The sequence was 2418 bp long and contained the *cyp512a3* gene full-length of 2119 bp, which consistsed of 11 exons.

ACCATTACACACGCAACCTTCCATACGCCCCAGAGTTGTCCGAGTTGGCAGCAGGCAT
GCGTCGCCATAAGTATCCTCTATCACCTTCCGCCTGTCTTGTTACTTCTCTCCCCTCTCA
ACCAGCTGGACCGTTGGACTCGTGCCCCATCTCTCTGATGGCAGTGGAGGATCCTCAA
GCTCTCATTCTCGCCGGCATCGCCATCCTCGCTGTCCTATACGTCGTCCGATGGCAGAC
AGACCCCGTAAGTGGGTCCTCACAAAGCGCTATGCGGTCAATGATAAACTAATTATCCG

AACAGCTGAGGTCTATCCCCACAGTTGGTGGGCCGTCCGCACCAGGACTATCGATGCT
GTCAGCGCTCAATTACCTGCGTAATGGGAAGAAGGTGATGACTGAGGGCTACCGGAAG
GTCTGTCTATCTTCTCCACCGGCAGCTCCGCAGGGACTCTGCAGGAGCTCACAAATCC
TGTTTAATAGTACCACGGAGGAGCCTTCAAGGTCGCACAGCTCGACAAATGGGTCGTC
GTGGTTTCCGGGCGCAAGTTGGTCGACGAACTCTGGCGGCGACCGGACGAGGATTTAT
CGGCTCCTGCGGCTGTTCAGGACGTACGAATACGCATGCAGCCGCACATCTTGTCCTAC
ATTGACTTGGTCTCCTTCGTAGATCATCCAGATGAGATATACTCTCGGACACGAAGCATT
TGATGACCCGTATGAAACCGACATCATCAAGGAGAAGCTTACGCGTTCGCTTCCGGTC
ATTTTTCCTGACGTCGTCGAGGAGATGAGGCTTGCCGTGTCGGACTACATCCCCACAA
AAGGAGACGGTAAGCAGTCTTTCAACCAGACCCTCTTTTGACGGGCTCTCAATTGAAT
TCACAAAGAGTGGACCCCTGTCAATGGTATGGACACGGCGGTGAATATTGTCGCAAGA
ACCAGTAGCCGCGCCTTCGTGGGTCTCCCACTTTGTAGGTCCTCGTTTTCACTTCATCA
GCATACCGACCAGCTCATGCGGAAACGCTTCAGGCCGCAACGAAGAATTCTTGGCGTT
GATTCTTCGTTTCACTATGGATGTGTTGAAGGATCGGTTCTTTCTCAACACTTTCCCGTA
CTTTTTGAAGCCGTGAGTCCAGTCTTGGAAAGTAATGACAGGATTTCTCACGTTCTCTG
GACAGTTACTTCGGGCACGCCTTCAGTAGAGCGAAGAGGACTATCTATCAAGGACTGA
CATTCCTCCAACCGTTGATCACCGAAAGAAGGATGAATATGCGAGAGCTTGGGGACGA
CTGGTCTGACAAGCCGGTAAGTGTCCCGTCGAGGGTCTGCGACAATGTGACTAAAGA
GTGCCTGTTGCAGGTTGATATGCTTCAGTGGGTCGTGGAAGCGGCGATGGCCAGGAAG
AGCGATGATTATAGGATTGCGGAACGCATGTTCCTCGTTAACTTCGCAGCTCTCCACAC
AACCTCCACCGTGCGCCCAATCTTCCCTGTCCTGCCAAGCGGTACTAATACCCTAGTTC
AACCCGTATAGACCCTTGCGCACGCGCTCTATGATCTCGCTGCCATGCCCGAGCTCATT
CCAGAACTGCGGGAAGAGGTCGATTCCGCCATCGCGTCGGACGGTTGGTCCAAAGCG
GCTGTAGGCAAAATGTGGAAGCTCGATAGCGTGTGCAGAGAGGCGTTGCGTTACCATG
GAATGAGCTTCAGTGTGTATCATATTATCTATTACACGAGCAGGCTCAGCGGTTCCCTGA
CTGACGGCCTACACCCCCCCCCTCCCACCAGTCGGTCTTTCCGCAAGGCAATGAAGG
ACGTCACGCTGAGCGACGGGACATTCATCCCGAAAGGCACCACCGTAGTCGCCGCAG
CAGGCCCCACGCACCACGAGGCGTCGATCTACCCCAACCCGGAGGTGCTCGACCCCTT
CCGCTTCGCAAAGTTGGGTGCAAGTGGCGGAGAAGGCGGTTCCGTGAAGCTCCAAAC
CGTTTCCACGTCCATTGATTTCCTGCCCTTCGGCCATGGCAAGCACGCTTGGTGTGTCC
CCTCTCCGCCACGCCCTGCTCGTGCCAAAGCAAGCGTTCTGACTGCCATACAGCCCGG
GACGGTGGTTCGCGGCAAACGAGGTGAAGATGATCTTAGCGCACATCGTGCTCAACTA
CGACCTGAAGCTCGGTGGAGACGGCAAACGGCCTGGGGACACGCTCTTCGGTACCAC
CATACTTCCGCCCCGTGGGCAGGTGTATTTCAGGAAACGCAAAGGGGTGCCCGAGTGA
GACAGAAGTTCCCGCTGATCGTTCGTCGCATCTCTGTAGGTTTTGAGCTGTTGTTGCTG
TATGTGGACTCGCTGTCTCGTCTTGGCGTTGATCTCCTTTTGCATTGTTCCTGTATCCCG
AAAGCCCACGAAGTGTAGAAGTCCG

**> Sequence amplified using primers *cyp5359n1*-F/ *cyp5359n1*-R.**

The sequence was 2059 bp long and contained the *cyp5359n1* gene full-length of 1968 bp, which consistsed of nine exons.

GCGCGGAAGGTATTTAGAGGACGTCCCCTTGCGCAGATGCTGGACACATCCCAAGCGC
ACCATGAACTGGACGCTTATCCTGACTTTTCTCTCTGCGGCGCTTTGTTACTCGCTGATA
TCTCTATCTCTTTGTTACAAGTTTTGCAAACAGGCTACTCGCTTCCGCTTCCGCCACCTC
CCTCCTGGTCCTCCCGCTTTACCTATCCTCGGGAACTTGTTCGATATACCCAAGACTCGC
CCATGGATAGGGTATCATGAGTTGAGTAAGAAGTACGGTAAGTTAGCTCTTCCTTTAGC
TGGTCATTGCAATCCCTTGACCACGAGATTAATAGGCAACTTGATGCTATTTCTCGTGCT
CGGTCAGTCTCTCGTCATCGTGAATGACGCTGGCACTGCGACGGATCTCCTTGAAGGG
CGCTCAACAAAGTACTCGTCCCGTCCAAGCTCAGTGCTAACGGAGCTGTAAGTGATCT
GCCATTGATACAGTGCCCAACTTGATGTCGTTTAGGCTGGGATGGGAGTGGAATATGGC
CTTCTTCCCGTACGGATGGTCGTGGCGTCAACATCGACGAGTTATTTGGAAGCACTACC
AACCAGACGCCATCCCCCGATACTATGAAGCGCAAACCCAGGCTGCTAGGAGACTACT
GGTCCTTTTGAGGTCCAACCCGGAGAAGCTGAGTGAGAACATTCGCTAGTAAGTCCGG
ACTAGACCTCCTCTATTTAGCCTCATGGCCTCACTAGAATTACCTTGCATACTCCCGGGT
TAGTGCTGTCGGTGCGACGCTCATTACGTCGACGTATGGTCTGGAAGTAGGCAGAGAC
CTCAGCGACAAGTATCTTTCTGCGTTCGAAACGGGTGTCAAGAGCGTCCGATTGTTTAT
TTCTGGATCGTCCATCCTCGAGTTCCTGCCGATTCTATCCCACGTTCCTACTTGGATGCC
TGGTGCTGGGTACCTTCGGGAATTGGTCGGCGTCCGGCGCACAACGCACCGGCTCCGA
GACTTGCCCTGGGGTGACGCAAGAGAAGCTGTTGTGAGTTCCTCAATTTCGAATCTAG
TATAGCTAACTGAGTGCGACTGAAAAATCGGTAGGTTAACGGGCATACAAATGGGCGA
GAAAGCATCGTTAGTGCAATGATCGAGGACTACGCTTATTTGAAGGACGACAGGCGGT
ATGCCTTCGAAGAGGAAGCAGCCAGAAATGTCGCCGCCGTGTCGTATGCAGGTACAGT
CGTTCCCTCATCAGGCGGTCTTTCGTATCCCTGACCATGATGTCGCTTTGTCAGCCGGA
ATAGATACGGTCAGAATTTCCTGCACTTGTCTGGTAGAACCACGATGCTCACTGGGCCC
TCAGACGCATGGCGTTCTATGCAATTTCTGCGTTGCCATGTCGCTTTATCCGGATGTACA
GAAGAAGGCCCAAGCCGAGCTGGACGCCGTCGTGGGGCCCCACCGCTTTCCCGAGTT
CAACGACCGCGACCATTTGCCATACGTCAACGCTGTCGTGAAGGAGATATTGCGATGG
AACCCGGTCACTCCGTTTGGACTCGCCCGTAAGACTACTTCGGATGACCACTACCATG
GGTACTTCATACCGGGAGGGAGCATCGTGATGGTAAACGTATGGTACGTCTCACCTGCG
CCATGCATTATGCCTCATTGCTTTCGTAGACTGACCCATGTGTCAATAGGTCAATTCTGA
ACGATCCCCAAGTCTACCCTGAACCGAACAAGTTCGTCCCAGAGAGGTTCTTGAAGGA
CGGCCGACTCAACCCGGGGGTCAAGGATCCTGCAGCCTACTTGTTCGGCTTTGGACGA
CGGTATGACAACCCCGATGCCCTTACATGTACAGAGCTCTGAGAACCTCCCCCTCGGAT
AGATTCTGCCCGGGACGCCACTTTGCGGACGCTTCATTGTTCATCCAGATAGCCTCCAT
GCTCCACTCGTTCAACATCGGTCCGCCTCTCGATGACAATGGTTCGCCGATCCACGTAA
TCCCACGGAGCGGTGACGGGCTTGTGTCGTAAGCCTCTCCAAGTAACGGCCATCGAGT
CTAC

**Supplementary Data S3:** Donor DNA sequences of *cyp512a3* and *cyp5359n1*. The sequence of 5' flank, *ura3* cassette and 3' flank was marked with green, red and blue letters, respectively.

**> Donor DNA sequence of *cyp512a3***

ATTTATGTCCCCCGTTCCTCATTCTCGCTCGATGGCATGATCAACCTCGCTTCCCAGGCT
TTACATCGGGGCATGCACGGTCATCCAGGGGCATGCGTGGCAACTTAAATCGACATGG
GCTTCCAGCAACCCGGCGAAAACAGACATTCCTGTGCGGTCTGAGCAAATGAAGCAA
GTGCCCTACCAAACCATCCTATATCTCGGAGGCGCGGTCACCTGGATCAGTAGATGTGA
GTAAGGACCATTACACACGCAACCTTCCATACGCCCCAGAGTTGTCCGAGTTGGCAGC
AGGCATGCGTCGCCATAAGTATCCTCTATCACCTTCCGCCTGTCTTGTTACTTCTCTCCC
CTCTCAACCAGCTGGACCGTTGGACTCGTGCCCCATCTCTCTGATGGCAGTGGAGGAT
CCTCAAGCTCTCATTCTCGCCGGCATCGCCATCCTCGCTGTCCTATACGTCGTCCGATGG
CAGACAGACCCCGTAAGTGGGTCCTCACAACACAGGTTCTTCAAGATGCTAGCCTCGG
ACGAGATGCGAGCCTGGTATGGTCCCGATCATGTCGCTCTTGCTTCAGATCGAGGCGCC
ATCGGCACACTTATGATCTCGGACGAGTTGTTCAGGTTAGCCCAGCTGACTCCTTCCGA
TGAACGTAGTCTTGATCATAATCCCAGGTCAAGCAACGCTACGGTGCGCAAAAAGTAC
GTGCGCATAGTGGAAGACGTCCAACAGAAAGGCGGGGAGGTCTTGATCTTCTCTAGTA
TGCACGAGTCTGGCCAGCGTAAGAGTTTTCCCATCCTGATGCTTACCGTACCACTGACA
TGGTCAATTTCATGCAGAGTTGAACCAACTAACGGGCATCGCTGCAATTTTGACCTTCC
CACTGGACATCGAAGTTGTAGAGGAAGAAGAGCGCGAAGCCAAAGAGGAAGAGGAG
AGAAGGAAGGCGGAAGTGGAAGGAACGACTGCCTGATTTAACGATAAGTATATGTATA
TGTATTGCGATTGTAGCGGTAGGATGTTTTGTTTACATATACACTGTGCTACTATACCCGT
CTCTGCACAATGCTAGCCCTAATCCGAGATCCCAACCCTTTCCTGGTATGCCTTCCAGC
CTTCTGCGCGGTACCTCTCCGCCTGTGCGCGGACTGCCTCCGCATTCGTGCCGTTATCG
TTCCCGTAAACACCCCGACCAACTATGATGACATCGCAATTGGACTCGATGACCACCTC
CCTTGGCGTTCGGTATTGCTGCCCCATGCTATCTCCCCTAGTATCCAGTCCGACTCCTGG
AGTAAGGATAAGGAAGTCTTCGTCCGGCGAGGACTCGCCCTCCCGCAGACCGACGCT
GTCCATGCGTTGCTGGGCAATGAAGCCGATCACGAAGTCGCGGTTGGCGCGAGCCATA
CGGACGGCCTCTTCCGTGTATGAGCCGGTTGCGAGGCTGCCCTTGGTGCTCATCTCGG
CGAGGAGCAGAAGCCCCCTGCCAAGGGGCAGACCGACAGCCTTGAGGCCGGAGATG
ATGGACGGTCCTGGGACAGGGTGTGCGTTCGTGATGTGCGACCAGCTAGCGATTTTGT
GCACGCCTGCAGAATATTGAAGTGCCACCGTGTTTCCTATATTCTTGTCAACATCGTATG
TAGCGGGCGCAAAGGGACTACATCCTACCAATGTCGGCAAATTTTCTGTCTTCAAAGAT
GAGGAAGTCATGCTTCTCGCTGAGTGCCTTGAGGCGTTCGATCGCGGATGGATCAAAG
TCCTCGATGATGTCTATGTGGGTCTTTGGATTATATTCACCCAAGTGCATATCAACCATTT
GGACATAGTACACCTACCTTTACTAGGCAGACGTATGGACCCACGATGTCCACAATCCT
GAAGAAGTCCTCCCGTTTCGTCACATCGACGCTAACAGACAGATTTGTGCGCTTGCGC
TCGATCGTCTCGAGGAGAGCTTTCGCGGCTGGGTTGGGATGTCTGGTGGCCCTCTGCG

CGTATGTTTGCTTGGCCACGGCCACCATTCTTTCAATTTTGTTTTGTGGCCGACGGGTG
GTTGCTGGGTGACTTGTGAGATTAGATAAGCGCGCTGTTTTCCAGTTTTGCCTCGAACG
AGGCTCTCCCATCTCTCCAGTGACCGTCTCTACTCCCCTCCGCTTCACTGACACCGAAT
GTCGTCCTCGCCAAAAACTCGCCCTCGCGAGCCAGATCTAGCGGATGAACCGAATGCC
AAGCGAGCAAAAGTAGAGGACGGGGACGGAGTCATGCCTATGAGCACGGACAGCACC
GTCACGGTTGTCAGCATCGCCGACGTCGAAGAGCAGGAACAGGACGATATGGACGTT
GACGTGAAGAACGACGAACCGGAGACTCTTCTTCCGCCAAGTCATGCGCTGCTCAAC
ACAAAACCCCCAGTGCATGGTCCAGATGGCTCTATACAGCAAATCATGGAAACGGACG
TTGGTATATCTGAGTACATTGGTTTTGATGTGCCCAAAATCGAGGGCATCATCAAGCAG
AGGTGCGTGGTAAGGTCATACTCGCGTTCGTTTATTTAGTTCATATGGCCTTCCAGGTTC
ACGGACTTCTTGGTATTCGAGGTCGACCAAGACAGTCAGGTCATCCATTTGAAGACGC
TCGCTATGCCATCCTCATCGAAGAACGGGGCGGATGTGACGCCTGCAGGAGTCTCCAC
TACGTCTAAGCCAGCTGATCAAGCCGTCGGTGAGAACAACGAGCCCGAAACTTCTGCC
ACCACTGCAGAGCAAGTTAGCGACGCTGTTGTCGCCGACCCCGCACCGGACGCCGCG
CAGCCACCCGCAACGGATGAACGAAAAGTTGACGGGGACACTCCACAAGAGGACGA
GCCATGGCCCGAACGCTTCACTGAATCACTCAAACCCTTCTTGTCAGAAGCCCTTATTG
CCCGTGTCAAGGACCTGTATCTTGAGGGACCAGAACCGCCATTCGTGAGTGACAGCGG
CTGGGCAGGCCGACAGGCGAAAATAGTTGGAGGGAGTGGAGACATTGACTTGGTCTC
CTTCGTAGATCATCCAGATGAGATATACTCTCGGACACGAAGCATTTGATGACCCGTAT
GAAACCGACATCATCAAGGAGAAGCTTACGCGTTCGCTTCCGGTCATTTTTCCTGACGT
CGTCGAGGAGATGAGGCTTGCCGTGTCGGACTACATCCCCACAAAAGGAGACGGTAA
GCAGTCTTTCAACCAGACCCTCTTTTGACGGGCTCTCAATTGAATTCACAAAGAGTGG
ACCCCTGTCAATGGTATGGACACGGCGGTGAATATTGTCGCAAGAACCAGTAGCCGCG
CCTTCGTGGGTCTCCCACTTTGTAGGTCCTCGTTTTCACTTCATCAGCATACCGACCAG
CTCATGCGGAAACGCTTCAGGCCGCAACGAAGAATTCTTGGCGTTGATTCTTCGTTTC
ACTATGGATGTGTTGAAGGATCGGTTCTTTCTCAACACTTTCCCGTACTTTTTGAAGCC
GTGAGTCCAGTCTTGGA

**> Donor DNA sequence of *cyp5359n1***

CGGGACTCATTTCGTGAGTGCCATGCCTCACAGTCCGATTGGTCGTGGCCAGCCTGATA
CTCTCTCAGATTCCTGGAGGACACCAGGTGCACACTCTCGCCGCGGTCGGCATGGGCA
GAGGCTCTAATCAGGCGCGAGTCGACAGACGCTGCCGTGTCCAACTGACCCTGGCCTT
TGGCTTCTCCTTGCGAGAAGCGGAGCCTTATGTGACTTAATGGAGACTGGAAAGGAAT
ATGAATGTCTTTTGCGGCATCGTGCTATCTCGGGCACCCGTTGAGGGGTAGGAGGTTCC
ATTCTCAACGACTCCAATCGCCTTTCGTGCATCCAAATCCAAGGTTCATCCGGTGTCAG
TTCCGCTTCTACACGGTGAATTGAAGAAGCCGTACCGTGGTCTGTGGCGCTGAGAGTA
TCGTTCTGTAGACTCTGGCCGCGTCTCACGGCGCGGAAGGTATTTAGAGGACGTCCCC
TTGCGCAGATGCTGGACACATCCCAAGCGCACCCACAGGTTCTTCAAGATGCTAGCCT

CGGACGAGATGCGAGCCTGGTATGGTCCCGATCATGTCGCTCTTGCTTCAGATCGAGGC
GCCATCGGCACACTTATGATCTCGGACGAGTTGTTCAGGTTAGCCCAGCTGACTCCTTC
CGATGAACGTAGTCTTGATCATAATCCCAGGTCAAGCAACGCTACGGTGCGCAAAAAG
TACGTGCGCATAGTGGAAGACGTCCAACAGAAAGGCGGGGAGGTCTTGATCTTCTCTA
GTATGCACGAGTCTGGCCAGCGTAAGAGTTTTCCCATCCTGATGCTTACCGTACCACTG
ACATGGTCAATTTCATGCAGAGTTGAACCAACTAACGGGCATCGCTGCAATTTTGACCT
TCCCACTGGACATCGAAGTTGTAGAGGAAGAAGAGCGCGAAGCCAAAGAGGAAGAG
GAGAGAAGGAAGGCGGAAGTGGAAGGAACGACTGCCTGATTTAACGATAAGTATATG
TATATGTATTGCGATTGTAGCGGTAGGATGTTTTGTTTACATATACACTGTGCTACTATAC
CCGTCTCTGCACAATGCTAGCCCTAATCCGAGATCCCAACCCTTTCCTGGTATGCCTTCC
AGCCTTCTGCGCGGTACCTCTCCGCCTGTGCGCGGACTGCCTCCGCATTCGTGCCGTTA
TCGTTCCCGTAAACACCCCGACCAACTATGATGACATCGCAATTGGACTCGATGACCAC
CTCCCTTGGCGTTCGGTATTGCTGCCCCATGCTATCTCCCCTAGTATCCAGTCCGACTCC
TGGAGTAAGGATAAGGAAGTCTTCGTCCGGCGAGGACTCGCCCTCCCGCAGACCGAC
GCTGTCCATGCGTTGCTGGGCAATGAAGCCGATCACGAAGTCGCGGTTGGCGCGAGCC
ATACGGACGGCCTCTTCCGTGTATGAGCCGGTTGCGAGGCTGCCCTTGGTGCTCATCTC
GGCGAGGAGCAGAAGCCCCCTGCCAAGGGGCAGACCGACAGCCTTGAGGCCGGAGA
TGATGGACGGTCCTGGGACAGGGTGTGCGTTCGTGATGTGCGACCAGCTAGCGATTTT
GTGCACGCCTGCAGAATATTGAAGTGCCACCGTGTTTCCTATATTCTTGTCAACATCGTA
TGTAGCGGGCGCAAAGGGACTACATCCTACCAATGTCGGCAAATTTTCTGTCTTCAAA
GATGAGGAAGTCATGCTTCTCGCTGAGTGCCTTGAGGCGTTCGATCGCGGATGGATCA
AAGTCCTCGATGATGTCTATGTGGGTCTTTGGATTATATTCACCCAAGTGCATATCAACC
ATTTGGACATAGTACACCTACCTTTACTAGGCAGACGTATGGACCCACGATGTCCACAA
TCCTGAAGAAGTCCTCCCGTTTCGTCACATCGACGCTAACAGACAGATTTGTGCGCTT
GCGCTCGATCGTCTCGAGGAGAGCTTTCGCGGCTGGGTTGGGATGTCTGGTGGCCCTC
TGCGCGTATGTTTGCTTGGCCACGGCCACCATTCTTTCAATTTTGTTTTGTGGCCGACG
GGTGGTTGCTGGGTGACTTGTGAGATTAGATAAGCGCGCTGTTTTCCAGTTTTGCCTCG
AACGAGGCTCTCCCATCTCTCCAGTGACCGTCTCTACTCCCCTCCGCTTCACTGACACC
GAATGTCGTCCTCGCCAAAAACTCGCCCTCGCGAGCCAGATCTAGCGGATGAACCGAA
TGCCAAGCGAGCAAAAGTAGAGGACGGGGACGGAGTCATGCCTATGAGCACGGACAG
CACCGTCACGGTTGTCAGCATCGCCGACGTCGAAGAGCAGGAACAGGACGATATGGA
CGTTGACGTGAAGAACGACGAACCGGAGACTCTTCTTCCGCCAAGTCATGCGCTGCTC
AACACAAAACCCCCAGTGCATGGTCCAGATGGCTCTATACAGCAAATCATGGAAACGG
ACGTTGGTATATCTGAGTACATTGGTTTTGATGTGCCCAAAATCGAGGGCATCATCAAG
CAGAGGTGCGTGGTAAGGTCATACTCGCGTTCGTTTATTTAGTTCATATGGCCTTCCAG
GTTCACGGACTTCTTGGTATTCGAGGTCGACCAAGACAGTCAGGTCATCCATTTGAAG
ACGCTCGCTATGCCATCCTCATCGAAGAACGGGGCGGATGTGACGCCTGCAGGAGTCT
CCACTACGTCTAAGCCAGCTGATCAAGCCGTCGGTGAGAACAACGAGCCCGAAACTT

CTGCCACCACTGCAGAGCAAGTTAGCGACGCTGTTGTCGCCGACCCCGCACCGGACG
CCGCGCAGCCACCCGCAACGGATGAACGAAAAGTTGACGGGGACACTCCACAAGAG
GACGAGCCATGGCCCGAACGCTTCACTGAATCACTCAAACCCTTCTTGTCAGAAGCCC
TTATTGCCCGTGTCAAGGACCTGTATCTTGAGGGACCAGAACCGCCATTCGTGAGTGA
CAGCGGCTGGGCAGGCCGACAGGCGAAAATAGTTGGAGGGAGTGGAGAACCCGGAG
AAGCTGAGTGAGAACATTCGCTAGTAAGTCCGGACTAGACCTCCTCTATTTAGCCTCAT
GGCCTCACTAGAATTACCTTGCATACTCCCGGGTTAGTGCTGTCGGTGCGACGCTCATT
ACGTCGACGTATGGTCTGGAAGTAGGCAGAGACCTCAGCGACAAGTATCTTTCTGCGT
TCGAAACGGGTGTCAAGAGCGTCCGATTGTTTATTTCTGGATCGTCCATCCTCGAGTTC
CTGCCGATTCTATCCCACGTTCCTACTTGGATGCCTGGTGCTGGGTACCTTCGGGAATT
GGTCGGCGTCCGGCGCACAACGCACCGGCTCCGAGACTTGCCCTGGGGTGACGCAAG
AGAAGCTGTTGTGAGTTCCTCAATTTCGAATCTAGTATAGCTAACTGAGTGCGACTGAA
AAATCGGTAGGTTAACGGGCATACAAATGGGCGAGAAAGCATCGTTAGTGCAATGATC
GAGGACTACGCTTATTTGAAGGA

**Supplementary Data S4:** Sequences of the four mutants edited through the *G. lucidum* CRISPR system. The *ura3* cassette sequences through homologous recombination (HR) were marked with red letters. The sequence introduced through non-homologous end joining (NHEJ) was marked with blue letters.

**> Sequence amplified in L1-Δ*cyp512a3.1* using primers *cyp512a3*-F/*cyp512a3*-R.**

ACCATTACACACGCAACCTTCCATACGCCCCAGAGTTGTCCGAGTTGGCAGCAGGCAT
GCGTCGCCATAAGTATCCTCTATCACCTTCCGCCTGTCTTGTTACTTCTCTCCCCTCTCA
ACCAGCTGGACCGTTGGACTCGTGCCCCATCTCTCTGATGGCAGTGGAGGATCCTCAA
GCTCTCATTCTCGCCGGCATCGCCATCCTCGCTGTCCTATACGTCATCCGATGGCAGAC
AGACCCCGTAAGTGGGTCCTCACAACACAGGTTCTTCAAGATGCTAGCCTCGGACGAG
ATGCGAGCCTGGTATGGTCCCGATCATGTCGCTCTTGCTTCAGATCGAGGCGCCATCGG
CACACCTATGATCTCGGACGAGTTGTTCAGGTTAGCCCAGCTGACTCCTTCCGATGAAC
GTAGTCTTGATCATAATCCCAGGTCAAGCAACGCTACGGTGCGCAAAAAGTACGTGCG
CATAGGGGAAGACGTCCAACAGAAAGGCGGGGAGGTCTTGATCTTCTCTAGTATGCAC
GAGTCTGGCCAGCGTAAGAGTTTTCCCATCCTGATGCTTACCGTACCACTGACATGGTC
AATTTCATGCAGAGTTGAACCAACTAACGGGCATCGCTGCAATTTTGACCTTCCCACTG
GACATCGAAGTTGTAGAGGAAGAAGAGCGCGAAGCCAAAGAGGAAGAGGAGAGAAG
GAAGGCGGAAGTGGAAGGAACGACTGCCTGATTTAACGATAAGTATATGTATATGTATT
GCGATTGTAGCGGTAGGATGTTTTGTTTACATATACACTGTGCTACTATACCCGTCTCTG
CACAATGCTAGCCCTAATCCGAGATCCCAACCCTTTCCTGGTATGCCTTCCAGCCTTCT
GCGCGGTACCTCTCCGCCTGTGCGCGGACTGCCTCCGCATTCGTGCCGTTATCGTTCCC
GTAAACACCCCGACCAACTATGATGACATCGCAATTGGACTCGATGACCACCTCCCTTG
GCGTTCGGTATTGCTGCCCCATGCTATCTCCCCTAGTATCCAGTCCGACTCCTGGAGTAA
GGATAAGGAAGTCTTCGTCCGGCGAGGACTCGCCCTCCCGCAGACCGACGCTGTCCAT
GCGTTGCTGGGCAATGAAGCCGATCACGAAGTCGCGGTTGGCGCGAGCCATACGGAC
GGCCTCTTCCGTGTATGAGCCGGTTGCGAGGCTGCCCTTGGTGCTCATCTCGGCGAGG
AGCAGAAGCCCCCTGCCAAGGGGCAGACCGACAGCCTTGAGGCCGGAGATGATGGAC
GGTCCTGGGACAGGGTGTGCGTTCGTGATGTGCGACCAGCTAGCGATTTTGTGCACGC
CTGCAGAATATTGAAGTGCCACCGTGTTTCCTATATTCTTGTCAACATCGTATGTAGCGG
GCGCAAAGGGACTACATCCTACCAATGTCGGCAAATTTTCTGTCTTCAAAGATGAGGA
AGTCATGCTTCTCGCTGAGTGCCTTGAGGCGTTCGATCGCGGATGGATCAAAGTCCTC
GATGATGTCTATGTGGGTCTTTGGATTATATTCACCCAAGTGCATATCAACCATTTGGAC
ATAGTACACCTACCTTTACTAGGCAGACGTATGGACCCACGATGTCCACAATCCTGAAG
AAGTCCTCCCGTTTCGTCACATCGACGCTAACAGACAGATTTGTGCGCTTGCGCTCGAT
CGTCTCGAGGAGAGCTTTCGCGGCTGGGTTGGGATGTCTGGTGGCCCTCTGCGCGTAT
GTTTGCTTGGCCACGGCCACCATTCTTTCAATTTTGTTTTGTGGCCGACGGGTGGTTGC
TGGGTGACTTGTGAGATTAGATAAGCGCGCTGTTTTCCAGTTTTGCCTCGAACGAGGCT
CTCCCATCTCTCCAGTGACCGTCTCTACTCCCCTCCGCTTCACTGACACCGAATGTCGT
CCTCGCCAAAAACTCGCCCTCGCGAGCCAGATCTAGCGGATGAACCGAATGCCAAGCG
AGCAAAAGTAGAGGACGGGGACGGAGTCATGCCTATGAGCACGGACAGCACCGTCAC
GGTTGTCAGCATCGCCGACGTCGAAGAGCAGGAACAGGACGATATGGACGTTGACGT
GAAGAACGACGAACCGGAGACTCTTCTTCCGCCAAGTCATGCGCTGCTCAACACAAA

ACCCCCAGTGCATGGTCCAGATGGCTCTATACAGCAAATCATGGAAACGGACGTTGGT
ATATCTGAGTACATTGGTTTTGATGTGCCCAAAATCGAGGGCATCATCAAGCAGAGGTG
CGTGGTAAGGTCATACTCGCGTTCGTTTATTTAGTTCATATGGCCTTCCAGGTTCACGGA
CTTCTTGGTATTCGAGGTCGACCAAGACAGTCAGGTCATCCATTTGAAGACGCTCGCC
ATGCCATCCTCATCGAAGAACGGGGCGGATGTGACGCCTGCAGGAGTCTCCACTACGT
CTAAGCCAGCTGATCAAGCCGTCGGTGAGAACAACGAGCCCGAAACTTCTGCCACCA
CTGCAGAGCAAGTTAGCGACGCTGTTGTCGCCGACCCCGCACCGGACGCCGCGCAGC
CACCCGCAACGGATGAACGAAAAGTTGACGGGGACACTCCACAAGAGGACGAGCCAT
GGCCCGAACGCTTCACTGAATCACTCAAACCCTTCTTGTCAGAAGCCCTTATTGCCCGT
GTCAAGGACCTGTATCTTGAGGGACCAGAACCGCCATTCGTGAGTGACAGCGGCTGG
GCAGGCCGACAGGCGAAAATAGTTGGAGGGAGTGGAGACATTGACTTGGTCTCCTTC
GTAGATCATCCAGATGAGATATACTCTCGGACACGAAGCATTTGATGACCCGTATGAAA
CCGACATCATCAAGGAGAAGCTTACGCGTTCGCTTCCGGTCATTTTTCCTGACGTCGTC
GAGGAGATGAGGCTTGCCGTGTCGGACTACATCCCCACAAAAGGAGACGGTAAGCAG
TCTTTCAACCAGACCCTCTTTTGACGGGCTCTCAATTGAATTCACAAAGAGTGGACCC
CTGTCAATGGTATGGACACGGCGGTGAATATTGTCGCAAGAACCAGTAGCCGCGCCTT
CGTGGGTCTCCCACTTTGTAGGTCCTCGTTTTCACTTCATCAGCATACCGACCAGCTCA
TGCGGAAACGCTTCAGGCCGCAACGAAGAATTCTTGGCGTTGATTCTTCGTTTCACTAT
GGATGTGTTGAAGGATCGGTTCTTTCTCAACACTTTCCCGTACTTTTTGAAGCCGTGAG
TCCAGTCTTGGAAAGTAATGACAGGATTTCTCACGTTCTCTGGACAGTTACTTCGGGCA
CGCCTTCAGTAGAGCGAAGAGGACTATCTATCAAGGACTGACATTCCTCCAACCGTTG
ATCACCGAAAGAAGGATGAATATGCGAGAGCTTGGGGACGACTGGTCTGACAAGCCG
GTAAGTGTCCCGTCGAGGGTCTGCGACAATGTGACTAAAGAGTGCCTGTTGCAGGTTG
ATATGCTTCAGTGGGTCGTGGAAGCGGCGATGGCCAGGAAGAGCGATGATTATAGGAT
TGCGGAACGCATGTTCCTCGTTAACTTCGCAGCTCTCCACACAACCTCCACCGTGCGC
CCAATCTTCCCTGTCCTGCCAAGCGGTACTAATACCCTAGTTCAACCCGTATAGACCCTT
GCGCACGCGCTCTATGATCTCGCTGCCATGCCCGAGCTCATTCCAGAACTGCGGGAAG
AGGTCGATTCCGCCATCGCGTCGGACGGTTGGTCCAAAGCGGCTGTAGGCAAAATGTG
GAAGCTCGATAGCGTGTGCAGAGAGGCGTTGCGTTACCATGGAATGAGCTTCAGTGTG
TATCATATTATCTATTACACGAGCAGGCTCAGCGGTTCCCTGACTGACGGCCTACACCCC
CCCCCTCCCACCAGTCGGTCTTTTCCGCAAGGCAATGAAGGACGTCACGCTGAGCGAC
GGGACATTCATCCCGAAAGGCACCACCGTAGTCGCCGCAGCAGGCCCCACGCACCAC
GAGGCGTCGATCTACCCCAACCCGGAGGTGCTCGACCCCTTCCGCTTCGCAAAGTTGG
GTGCAAGTGGCGGAGAAGGCGGTTCCGTGAAGCTCCAAACCGTTTCCACGTCCATTG
ATTTCCTGCCCTTCGGCCATGGCAAGCACGCTTGGTGTGTCCCCTCTCCGCCACGCCCT
GCTCGTGCCAAAGCAAGCGTTCTGACTGCCATACAGCCCGGGACGGTGGTTCGCGGC
AAACGAGGTGAAGATGATCTTAGCGCACATCGTGCTCAACTACGACCTGAAGCTCGGT
GGAGACGGCAAACGGCCTGGGGACACGCTCTTCGGTACCACCATACTTCCGCCCCGTG
GGCAGGTGTATTTCAGGAAACGCAAAGGGGTGCCCGAGTGAGACAGAAGTTCCCGCT
GATCGTTCGTCGCATCTCTGTAGGTTTTGAGCTGTTGTTGCTGTATGTGGACTCGCTGTC
TCGTCTTGGCGTTGATCTCCTTTTGCATTGTTCCTGTATCCCGAAAGCCCACGAAGTGT
AGAAGTCCG

**> Sequence amplified in L1-Δ*cyp512a3.2* using primers *cyp512a3*-F/*cyp512a3*-R.**

ACCATTACACACGCAACCTTCCATACGCCCCAGAGTTGTCCGAGTTGGCAGCAGGCAT
GCGTCGCCATAAGCATCCTCTATCACCTTCCGCCTGTCTTGTTACTTCTCTCCCCTCTCA
ACCAGCTGGACCGTTGGACTCGTGCCCCATCTCCCTGATGGCAGTGGAGGATCCTCAA
GCTCTCATTCTCGCCGGCATCGCCATCCTCGCTGTCCTATACGTCGTCCGATGGCAGAC
AGACCCCGTAAGTGGTCCTCACAACACAGGTTCTTCAAGATGCTAGCCTCGGACGAGA
TGCGAGCCTGGTATGGTCCCGATCACGTCGCTCTTGCTTCAGATCGAGGCGCCATCGGC
ACACTTATGATCTCGGACGAGTTGTTCAGGTTAGCCCAGCTGACTCCTTCCGATGAACG
TAGTCTTGATCATAATCCCAGGTCAAGCAACGCTACGGTGCGCAAAAAGTACGTGCGC
ATAGTGGAAGACGTCCAACAGAAAGGCGGGGAGGTCTCGATCTTCTCTAGTATGCACG
AGTCTGGCCAGCGTAAGAGTTTTCCCATCCTGATGCTTACCGTACCACTGACATGGTCA
ATTTCATGCAGAGTTGAACCAACTAACGGGCATCGCTGCAATTTTGACCTTCCCACTGG
ACATCGAAGTTGTAGAGGAAGAAGAGCGCGAAGCCAAAGAGGAAGAGGAGAGAAGG
AAGGCGGAAGTGGAAGGAACGACAGCCTGATTTAACGATAAGTATATGTATATGTATTG
CGATTGTAGCGGTAGGATGTTTTGTTTACATATACACTGTGCTACTATACCCGTCTCTGC
ACAATGCTAGCCCTAATCCGAGATCCCAACCCTTTCCTGGTATGCCTTCCAGCCTTCTG
CGCGGTACCTCTCCGCCTGTGCGCGGACTGCCTCCGCATTCGTGCCGTTATCGTTCCCG
TAAACACCCCGACCAACTATGATGACATCGCAATTGGACTCGATGACCACCTCCCTTGG
CGTTCGGTATTGCTGCCCCATGCTATCTCCCCTAGTATCCAGTCCGACTCCTGGAGTAAG
GATAAGGAAGTCTTCGTCCGGCGAGGACTCGCCCTCCCGCAGACCGACGCTGTCCATG
CGTTGCTGGGCAATGAAGCCGATCACGAAGTCGCGGTTGGCGCGAGCCATACGGACG
GCCTCTTCCGTGTATGAGCCGGTTGCGAGGCTGCCCTTGGTGCTCATCTCGGCGAGGA
GCAGAAGCCCCCTGCCAAGGGGCAGACCGACAGCCTTGAGGCCGGAGATGATGGACG
GTCCTGGGACAGGGTGTGCGTTCGTGATGTGCGACCAGCTAGCGATTTTGTGCACGCC
TGCAGAATATTGAAGTGCCACCGTGTTTCCTATATTCTTGTCAACATCGTATGTAGCGGG
CGCAAAGGGACTACATCCTACCAATGTCGGCAAATTTTCTGTCTTCAAAGATGAGGAA
GTCATGCTTCTCGCTGAGTGCCTTGAGGCGTTCGATCGCGGATGGATCAAAGTCCTCG
ATGATGTCTATGTGGGTCTTTGGATTATATTCACCCAAGTGCATATCAACCATTTGGACA
TAGTACACCTACCTTTACTAGGCAGACGTATGGACCCACGATGTCCACAATCCTGAAGA
AGTCCTCCCGTTTCGTCACATCGACGCAAACAGACAGATTTGTGCGCTTGCGCTCGAT
CGTCTCGAGGAGAGCTTTCGCGGCTGGGTTGGGATGTCTGGTGGCCCTCTGCGCGTAT
GTTTGCTTGGCCACGGCCACCATTCTTTCAATTTTGTTTTGTGGCCGACGGGTGGTTGC
TGGGTGACTTGTGAGATTAGATAAGCGCGCTGTTTTCCAGTTTTGCCTCGAACGAGGCT
CTCCCATCTCTCCAGTGACCGTCTCTACTCCCCTCCGCTTCACTGACACCGAATGTCGT
CCTCGCCAAAAACTCGCCCTCGCGAGCCAGATCTAGCGGATGAACCGAATGCCAAGCG
AGCAAAAGTAGAGGACGGGGACGGAGTCATGCCTATGAGCACGGACAGCACCGTCAC
GGTTGTCAGCATCGCCGACGTCGAAGAGCAGGAACAGGACGATATGGACGTTGACGT
GAAGAACGACGAACCGGAGACTCTTCTTCCGCCAAGTCATGCGCTGCTCAACACAAA
ACCCCCAGTGCATGGTCCAGATGGCTCTATACAGCAAATCATGGAAACGGACGTTGGT
ATATCTGAGTACATTGGTTTTGATGTGCCCAAAATCGAGGGCATCATCAAGCAGAGGTG
CGTGGTAAGGTCATACTCGCGTTCGTTTATTTAGTTCATATGGCCTTCCAGGTTCACGGA
CTTCTTGGTATTCGAGGTCGACCAAGACAGTCAGGTCATCCATTTGAAGACGCTCGCTA
TGCCATCCTCATCGAAGAACGGGGCGGATGTGACGCCTGCAGGAGTCTCCACTACGTC
TAAGCCAGCTGATCAAGCCGTCGGTGAGAACAACGAGCCCGAAACTTCTGCCACCGC
TGCAGAGCAAGTTAGCGACGCTGTTGTCGCCGACCCCGCACCGGACGCCGCGCAGCC

ACCCGCAACGGATGAACGAAAAGTTGACGGGGACACTCCACAAGAGGACGAGCCAT
GGCCCGAACGCTTCACTGAATCACTCAAACCCTTCTTGTCAGAAGCCCTTATTGCCCGT
GTCAAGGACCTGTATCTTGAGGGACCAGAACCGCCATTCGTGAGTGACAGCGGCTGG
GCAGGCCGACAGGCGAAAATAGTTGGAGGGAGTGGAGACATTGACTTGGTCTCCTTC
GTAGATCATCCAGATGAGATATACTCTCGGACACGAAGCATCTGATGACCCGTATGAAA
CCGACATCATCAAGGAGAAGCTTACGCGTTCGCTTCCGGTCATTTTTCCTGACGTCGTC
GAGGAGATGAGGCTTGCCGTGTCGGACTACATCCCCACAAAAGGAGACGGTAAGCAG
TCTTTCAACCAGACCCTCTTTTGACGGGCTCTCAATTGAATTCACAAAGAGTGGACCC
CTGTCAATGGTATGGACACGGCGGTGAATATTGTCGCAAGAACCAGTAGCCGCGCCTT
CGTGGGTCTCCCACTTTGTAGGTCCTCGTTTTCACTTCATCAGCATACCGACCAGCTCA
TGCGGAAACGCTTCAGGCCGCAACGAAGAATTCTTGGCGTTGATTCTTCGTTTCACTAT
GGATGTGTTGAAGGATCGGTTCTTTCTCAACACTTTCCCGTACTTTTTGAAGCCGTGAG
TCCAGTCTTGGAAAGTAATGACAGGATTTCTCACGTTCTCTGGACAGTTACTTCGGGCA
CGCCTTCAGTAGAGCGAAGAGGACTATCTATCAAGGACTGACATTCCTCCAACCGTTG
ATCACCGAAAGAAGGATGAATATGCGAGAGCTTGGGGACGACTGGTCTGACAAGCCG
GTAAGTGTCCCGTCGAGGGTCTGCGACAATGTGACTAAAGAGTGCCTGTTGCAGGTTG
ATATGCTTCAGTGGGTCGTGGAAGCGGCGATGGCCAGGAAGAGCGATGATTATAGGAT
TGCGGAACGCATGTTCCTCGTTAACTTCGCAGCTCTCCACACAACCTCCACCGTGCGC
CCAATCTTCCCTGTCCTGCCAAGCGGTACTAATACCCTAGTTCAACCCGTATAGACCCTT
GCGCACGCGCTCTATGATCTCGCTGCCATGCCCGAGCTCATTCCAGAACTGCGGGAAG
AGGTCGATTCCGCCATCGCGTCGGACGGTTGGTCCAAAGCGGCTGTAGGCAAAATGTG
GAAGCTCGATAGCGTGTGCAGAGAGGCGTTGCGTTACCATGGAATGAGCTTCAGTGTG
TATCATATTATCTATTACACGAGCAGGCTCAGCGGTTCCCTGACTGACGGCCTACACCCC
CCCCCTCCCACCAGTCGGTCTTTTCCGCAAGGCAATGAAGGACGTCACGCTGAGCGAC
GGGACATTCATCCCGAAAGGCACCACCGTAGTCGCCGCAGCAGGCCCCACGCACCAC
GAGGCGTCGATCTACCCCAACCCGGAGGTGCTCGACCCCTTCCGCTTCGCAAAGTTGG
GTGCAAGTGGCGGAGAAGGCGGTTCCGTGAAGCTCCAAACCGTTTCCACGTCCATTG
ATTTCCTGCCCTTCGGCCATGGCAAGCACGCTTGGTGTGTCCCCTCTCCGCCACGCCCT
GCTCGTGCCAAAGCAAGCGTTCTGACTGCCATACAGCCCGGGACGGTGGTTCGCGGC
AAACGAGGTGAAGATGATCTTAGCGCACATCGTGCTCAACTACGACCTGAAGCTCGGT
GGAGACGGCAAACGGCCTGGGGACACGCTCTTCGGTACCACCATACTTCCGCCCCGTG
GGCAGGTGTATTTCAGGAAACGCAAAGGGGTGCCCGAGTGAGACAGAAGTTCCCGCT
GATCGTTCGTCGCATCTCTGTAGGTTTTGAGCTGTTGTTGCTGTATGTGGACTCGCTGTC
TCGTCTTGGCGTTGATCTCCTTTTGCATTGTTCCTGTATCCCGAAAGCCCACGAAGTGT
AGAAGTCCG

**> Sequence amplified in L1-Δ*cyp5359n1.1* using primers *cyp5359n1*-F/ *cyp5359n1*-R.**

GCGCGGAAGGTATTTAGAGGACGTCCCCTTGCGCAGATGCTGGACACATCCCAAGCGC
ACCCACAGGTTCTTCAAGATGCTAGCCTCGGACGAGATGCGAGCCTGGTATGGTCCCG
ATCATGTCGCTCTTGCTTCAGATCGAGGCGCCATCGGCACACTTATGATCTCGGACGAG
TTGTTCAGGTTAGCCCAGCTGACTCCTTCCGATGAACGTAGTCTTGATCATAATCCCAG
GTCAAGCGACGCTACGGTGCGCAAAAAGTACGTGCGCATAGTGGAAGACGTCCAACA
GAAAGGCGGGGAGGTCTTGATCTTCTCTAGTATGCACGAGTCTGGCCAGCGTAAGAGT
TTTCCCATCCTGATGCTTACCGTACCACTGACATGGTCAATTTCATGCAGAGTTGAACC

AACTAACGGGCATCGCTGCAATTTTGACCTTCCCACTGGACATCGAAGTTGTAGAGGA
AGAAGAGCGCGAAGCCAAAGAGGAAGAGGAGAGAAGGAAGGCGGAAGTGGAAGGA
GCGACTGCCTGATTTAACGATAAGTATATGTATATGTATTGCGATTGTAGCGGTAGGATG
TTTTGTTTACATATACACTGTGCTACTATACCCGTCTCTGCACAATGCTAGCCCTAATCCG
AGATCCCAACCCTTTCCTGGTATGCCTTCCAGCCTTCTGCGCGGTACCTCTCCGCCTGT
GCGCGGACTGCCTCCGCATTCGTGCCGTTATCGTTCCCGTAAACACCCCGACCAACTAT
GATGACATCGCAATTGGACTCGATGACCACCTCCCTTGGCGTTCGGTATTGCTGCCCCA
TGCTATCTCCCCTAGTATCCAGTCCGACTCCTGGAGTAAGGATAAGGAAGTCTTCGTCC
GGCGAGGACTCGCCCTCCCGCAGACCGACGCTGTCCATGCGTTGCTGGGCAATGAAG
CCGATCACGAAGTCGCGGTTGGCGCGAGCCATACGGACGGCCTCTTCCGTGTATGAGC
CGGTTGCGAGGCTGCCCTTGGTGCTCATCTCGGCGAGGAGCAGAAGCCCCCTGCCAA
GGGGCAGACCGACAGCCTTGAGGCCGGAGATGATGGACGGTCCTGGGACAGGGTGTG
CGTTCGTGATGTGCGACCAGCTAGCGATTTTGTGCACGCCTGCAGAATATTGAAGTGCC
ACCGTGTTTCCTATATTCTTGTCAACATCGTATGTAGCGGGCGCAAAGGGACTACATCCT
ACCAATGTCGGCAAATTTTCTGTCTTCAAAGATGAGGAAGTCATGCTTCTCGCTGAGTG
CCTTGAGGCGTTCGATCGCGGATGGATCAAAGTCCTCGATGATGTCTATGTGGGTCTTT
GGATTATATTCACCCAAGTGCATATCAACCATTTGGACATAGTACACCTACCTTTACTAG
GCAGACGTATGGACCCACGATGTCCACAATCCTGAAGAAGTCCTCCCGTTTCGTCACA
TCGACGCTAACAGACAGATTTGTGCGCTTGCGCTCGATCGTCTCGAGGAGAGCTTTCG
CGGCTGGGTTGGGATGTCTGGTGGCCCTCTGCGCGTATGTTTGCTTGGCCACGGCCAC
CATTCTTTCAATTTTGTTTTGTGGCCGACGGGTGGTTGCTGGGTGACTTGTGAGATTAG
ATAAGCGCGCTGTTTTCCAGTTTTGCCTCGAACGAGGCTCTCCCATCTCTCCAGTGACC
GTCTCTACTCCCCTCCGCTTCACTGACACCGAATGTCGTCCTCGCCAAAAACTCGCCCT
CGCGAGCCAGATCTAGCGGATGAACCGAATGCCAAGCGAACAAAAGTAGAGGACGGG
GACGGAGTCATGCCTATGAGCACGGACAGCACCGTCACGGTTGTCAGCATCGCCGACG
TCGAAGAGCAGGAACAGGACGATATGGACGTTGACGTGAAGAACGACGAACCGGAG
ACTCTTCTTCCGCCAAGTCATGCGCTGCTCAACACAAAACCCCCAGTGCATGGTCCAG
ATGGCTCTATACAGCAAATCATGGAAACGGACGTTGGTATATCTGAGTACATTGGTTTG
ATGTGCCCAAAATCGAGGGCATCATCAAGCAGAGGTGCGTGGTAAGGTCATACTCGCG
TTCGTTTATTTAGTTCATATGGCCTTCCAGGTTCACGGACTTCTTGGTATTCGAGGTCGA
CCAAGACAGTCAGGTCATCCATTTGAAGACGCTCGCTATGCCATCCTCATCGAAGAAC
GGGGCGGATGTGACGCCTGCAGGAGTCTCCACTACGTCTAAGCCAGCTGATCAAGCCG
TCGGTGAGAACAACGAGCCCGAAACTTCTGCCACCACTGCAGAGCAAGTTAGCGACG
CTGTTGTCGCCGACCCCGCACCGGACGCCGCGCAGCCACCCGCAACGGATGAACGAA
AAGTTGACGGGGACACTCCACAAGAGGACGAGCCATGGCCCGAACGCTTCACTGAAT
CACTCAAACCCTTCTTGTCAGAAGCCCTTATTGCCCGTGTCAAGGACCTGTATCTTGAG
GGACCAGAACCGCCATTCGTGAGTGACAGCGGCTGGGCAGGCCGACAGGCGAAAATA
GTTGGAGGGAGTGGAGAACCCGGAGAAGCTGAGTGAGAACATTCGCTAGTAAGTCCG
GACTAGACCTCCTCTATTTAGCCTCATGGCCTCACTAGAATTACCTTGCATACTCCCGGG
TTAGTGCTGTCGGTGCGACGCTCATTACGTCGACGTATGGTCTGGAAGTAGGCAGAGA
CCTCAGCGACAAGTATCTTTCTGCGTTCGAAACGGGTGTCAAGAGCGTCCGATTGTTT
ATTTCTGGATCGTCCATCCTCGAGTTCCTGCCGATTCTATCCCACGTTCCTACTTGGATG
CCTGGTGCTGGGTACCTTCGGGAATTGGTCGGCGTCCGGCGCACAACGCACCGGCTCC
GAGACTTGCCCTGGGGTGACGCAAGAGAAGCTGTTGTGAGTTCCTCAATTTCGAATCT

AGTATAGCTAACTGAGTGCGACTGAAAAATCGGTAGGTTAACGGGCATACAAATGGGC
GAGAAAGCATCGTTAGTGCAATGATCGAGGACTACGCTTATTTGAAGGACGACAGGCG
GTATGCCTTCGAAGAGGAAGCAGCCAGAAATGTCGCCGCCGTGTCGTATGCAGGTACA
GTCGTTCCCTCATCAGGCGGTCTTTCGTATCCCTGACCATGATGTCGCTTTGTCAGCCG
GAATAGATACGGTCAGAATTTCCTGCACTTGTCTGGTAGAACCACGATGCTCACTGGGC
CCTCAGACGCATGGCGTTCTATGCAATTTCTGCGTTGCCATGTCGCTTTATCCGGATGTA
CAGAAGAAGGCCCAAGCCGAGCTGGACGCCGTCGTGGGGCCCCACCGCTTTCCCGAG
TTCAACGACCGCGACCATTTGCCATACGTCAACGCTGTCGTGAAGGAGATATTGCGATG
GAACCCGGTCACTCCGTTTGGACTCGCCCGTAAGACTACTTCGGATGACCACTACCAT
GGGTACTTCATACCGGGAGGGAGCATCGTGATGGTAAACGTATGGTACGTCTCACCTGC
GCCATGCATTATGCCTCATTGCTTTCGTAGACTGACCCATGTGTCAATAGGTCAATTCTG
AACGATCCCCAAGTCTACCCTGAACCGAACAAGTTCGTCCCAGAGAGGTTCTTGAAGG
ACGGCCGACTCAACCCGGGGGTCAAGGATCCTGCAGCCTACTTGTTCGGCTTTGGACG
ACGGTATGACAACCCCGATGCCCTTACATGTACAGAGCTCTGAGAACCTCCCCCTCGG
ATAGATTCTGCCCGGGACGCCACTTTGCGGACGCTTCATTGTTCATCCAGATAGCCTCC
ATGCTCCACTCGTTCAACATCGGTCCGCCTCTCGATGACAATGGTTCGCCGATCCACGT
AATCCCACGGAGCGGTGACGGGCTTGTGTCGTAAGCCTCTCCAAGTAACGGCCATCGA
GTCTAC

**> Sequence amplified in L1-Δ*cyp5359n1.2* using primers *cyp5359n1*-F/ *cyp5359n1*-R.**

GCGCGGAAGGTATTTAGAGGACGTCCCCTTGCGCAGATGCTGGACACATCCCAAGCGC
ACCCACAGGTTCTTCAAGATGCTAGCCTCGGACGAGATGCGAGCCTGGTATGGTCCCG
ATCATGTCGCTCTTGCTTCAGATCGAGGCGCCATCGGCACACTTATGATCTCGGACGAG
TTGTTCAGGTTAGCCCAGCTGACTCCTTCCGATGAACGTAGTCTTGATCATAATCCCAG
GTCAAGCGACGCTACGGTGCGCAAAAAGTACGTGCGCATAGTGGAAGACGTCCAACA
GAAAGGCGGGGAGGTCTTGATCTTCTCTAGTATGCACGAGTCTGGCCAGCGTAAGAGT
TTTCCCATCCTGATGCTTACCGTACCACTGACATGGTCAATTTCATGCAGAGTTGAACC
AACTAACGGGCATCGCTGCAATTTTGACCTTCCCACTGGACATCGAAGTTGTAGAGGA
AGAAGAGCGCGAAGCCAAAGAGGAAGAGGAGAGAAGGAAGGCGGAAGTGGAAGGA
GCGACTGCCTGATTTAACGATAAGTATATGTATATGTATTGCGATTGTAGCGGTAGGATG
TTTTGTTTACATATACACTGTGCTACTATACCCGTCTCTGCACAATGCTAGCCCTAATCCG
AGATCCCAACCCTTTCCTGGTATGCCTTCCAGCCTTCTGCGCGGTACCTCTCCGCCTGT
GCGCGGACTGCCTCCGCATTCGTGCCGTTATCGTTCCCGTAAACACCCCGACCAACTAT
GATGACATCGCAATTGGACTCGATGACCACCTCCCTTGGCGTTCGGTATTGCTGCCCCA
TGCTATCTCCCCTAGTATCCAGTCCGACTCCTGGAGTAAGGATAAGGAAGTCTTCGTCC
GGCGAGGACTCGCCCTCCCGCAGACCGACGCTGTCCATGCGTTGCTGGGCAATGAAG
CCGATCACGAAGTCGCGGTTGGCGCGAGCCATACGGACGGCCTCTTCCGTGTATGAGC
CGGTTGCGAGGCTGCCCTTGGTGCTCATCTCGGCGAGGAGCAGAAGCCCCCTGCCAA
GGGGCAGACCGACAGCCTTGAGGCCGGAGATGATGGACGGTCCTGGGACAGGGTGTG
CGTTCGTGATGTGCGACCAGCTAGCGATTTTGTGCACGCCTGCAGAATATTGAAGTGCC
ACCGTGTTTCCTATATTCTTGTCAACATCGTATGTAGCGGGCGCAAAGGGACTACATCCT
ACCAATGTCGGCAAATTTTCTGTCTTCAAAGATGAGGAAGTCATGCTTCTCGCTGAGTG
CCTTGAGGCGTTCGATCGCGGATGGATCAAAGTCCTCGATGATGTCTATGTGGGTCTTT
GGATTATATTCACCCAAGTGCATATCAACCATTTGGACATAGTACACCTACCTTTACTAG

GCAGACGTATGGACCCACGATGTCCACAATCCTGAAGAAGTCCTCCCGTTTCGTCACA
TCGACGCTAACAGACAGATTTGTGCGCTTGCGCTCGATCGTCTCGAGGAGAGCTTTCG
CGGCTGGGTTGGGATGTCTGGTGGCCCTCTGCGCGTATGTTTGCTTGGCCACGGCCAC
CATTCTTTCAATTTTGTTTTGTGCTATGAAGCGCAAACCCAGGCTGCTAGGAGACTACT
GGTCCTTTTGAGGTCCAACCCGGAGAAGCTGAGTGAGAACATTCGCTAGTAAGTCCGG
ACTAGACCTCCTCTATTTAGCCTCATGGCCTCACTAGAATTACCTTGCATACTCCCGGGT
TAGTGCTGTCGGTGCGACGCTCATTACGTCGACGTATGGTCTGGAAGTAGGCAGAGAC
CTCAGCGACAAGTATCTTTCTGCGTTCGAAACGGGTGTCAAGAGCGTCCGATTGTTTAT
TTCTGGATCGTCCATCCTCGAGTTCCTGCCGATTCTATCCCACGTTCCTACTTGGATGCC
TGGTGCTGGGTACCTTCGGGAATTGGTCGGCGTCCGGCGCACAACGCACCGGCTCCGA
GACTTGCCCTGGGGTGACGCAAGAGAAGCTGTTGTGAGTTCCTCAATTTCGAATCTAG
TATAGCTAACTGAGTGCGACTGAAAAATCGGTAGGTTAACGGGCATACAAATGGGCGA
GAAAGCATCGTTAGTGCAATGATCGAGGACTACGCTTATTTGAAGGACGACAGGCGGT
ATGCCTTCGAAGAGGAAGCAGCCAGAAATGTCGCCGCCGTGTCGTATGCAGGTACAGT
CGTTCCCTCATCAGGCGGTCTTTCGTATCCCTGACCATGATGTCGCTTTGTCAGCCGGA
ATAGATACGGTCAGAATTTCCTGCACTTGTCTGGTAGAACCACGATGCTCACTGGGCCC
TCAGACGCATGGCGTTCTATGCAATTTCTGCGTTGCCATGTCGCTTTATCCGGATGTACA
GAAGAAGGCCCAAGCCGAGCTGGACGCCGTCGTGGGGCCCCACCGCTTTCCCGAGTT
CAACGACCGCGACCATTTGCCATACGTCAACGCTGTCGTGAAGGAGATATTGCGATGG
AACCCGGTCACTCCGTTTGGACTCGCCCGTAAGACTACTTCGGATGACCACTACCATG
GGTACTTCATACCGGGAGGGAGCATCGTGATGGTAAACGTATGGTACGTCTCACCTGCG
CCATGCATTATGCCTCATTGCTTTCGTAGACTGACCCATGTGTCAATAGGTCAATTCTGA
ACGATCCCCAAGTCTACCCTGAACCGAACAAGTTCGTCCCAGAGAGGTTCTTGAAGGA
CGGCCGACTCAACCCGGGGGTCAAGGATCCTGCAGCCTACTTGTTCGGCTTTGGACGA
CGGTATGACAACCCCGATGCCCTTACATGTACAGAGCTCTGAGAACCTCCCCCTCGGAT
AGATTCTGCCCGGGACGCCACTTTGCGGACGCTTCATTGTTCATCCAGATAGCCTCCAT
GCTCCACTCGTTCAACATCGGTCCGCCTCTCGATGACAATGGTTCGCCGATCCACGTAA
TCCCACGGAGCGGTGACGGGCTTGTGTCGTAAGCCTCTCCAAGTAACGGCCATCGAGT
CTAC

**Table S1.** Primer sequences

| Primer | Sequence (5'→3') |
|---|---|
| *ura3*-F | AATCTCACAAGTCACCCAGC |
| *ura3*-R | GCGATTGTAGCGGTAGGATG |
| *cyp512a3*-F | ACCATTACACACGCAACCTT |
| *cyp512a3*-R | CGGACTTCTACACTTCGTGG |
| *cyp5359n1*-F | GCGCGGAAGGTATTTAGAGG |
| *cyp5359n1*-R | GTAGACTCGATGGCCGTTAC |
| *cyp512a3*-Up-F | ATTTATGTCCCCCGTTCCTCATTCTCG |
| *cyp512a3*-Up-R | GAGGCTAGCATCTTGAAGAACCTGTGTTGTGAGGACCCACTTACGGGGT |
| *cyp512a3*-Down-F | GCGAAAATAGTTGGAGGGAGTGGAGACATTGACTTGGTCTCCTTCGTAGATCAT |
| *cyp512a3*-Down-R | TCCAAGACTGGACTCACGGCTTC |
| *ura3*-*cyp512a3*-F | ACCCCGTAAGTGGGTCCTCACAACACAGGTTCTTCAAGATGCTAGCCTC |
| *ura3*-*cyp512a3*-R | ATGATCTACGAAGGAGACCAAGTCAATGTCTCCACTCCCTCCAACTATTTTCGC |
| *cyp5359n1*-Up-F | CGGGACTCATTTCGTGAGTGCC |
| *cyp5359n1*-Up-R | GGCTAGCATCTTGAAGAACCTGTGGGTGCGCTTGGGATGTGTC |
| *cyp5359n1*-Down-F | CGAAAATAGTTGGAGGGAGTGGAGAACCCGGAGAAGCTGAGTGAGA |
| *cyp5359n1*-Down-R | TCCTTCAAATAAGCGTAGTCCTCGATC |
| *ura3*-*cyp5359n1*-F | GACACATCCCAAGCGCACCCACAGGTTCTTCAAGATGCTAGCC |
| *ura3*-*cyp5359n1*-R | TCTCACTCAGCTTCTCCGGGTTCTCCACTCCCTCCAACTATTTTCG |
| E-*ura3*-F | CACTCAGCGAGAAGCATGAC |
| E-*ura3*-R | ACAATGCTAGCCCTAATCCGA |

**Table S2.** Source analysis of newly introduced sequences in 35 mutants.

| No. | Mutants | Mutation Type | Newly Introduced Sequences of L1 Genome | Whether the Introduced Sequences from L1 Genome Itself |
|---|---|---|---|---|
| 1 | RNP73.5-8 | Insertion of 112 bp | TGTCGCGTTAGAGCGACTGTAGGCGACTGTGCATGATGCATCGG GCACCGCAGGACGCTGACTCCAGAATTTCATGATAGCTTTACGG GATGCATGCAAGCTCTGCGCTCCT | Yes |
| 2 | RNP73.5-9 | Substitutions of 65 bp to 10 bp | ATTAACACTA | Yes |
| 3 | RNP73.5-10 | Substitutions of 1 bp to 72 bp | GAGGAGTTAATAAAGATAAGTCTAAAGGAACTATTTTTACTTTA TCCTTATAAAACTTTAAATAAAACTCAT | No |
| 4 | RNP147-9 | Insertion of 32 bp | AACCAGTGGAAGGTCGTCATTTCCGAGGAGCG | Yes |
| 5 | RNP147-10 | Insertion of 106 bp | GACTCGCAAACCGTCCGACTATAGGCGGGCACTCATCGCGGTC CACACCGCAGTAATCTACTAATAGTGACGGGTGTGACAGTCTA GAAGCTGGCCAGCGGAGACA | Yes |
| 6 | RNP220.6-19 | Insertion of 28 bp | ATCGGGTTCCTCTTATCGTCAAACTTAT | No |
| 7 | RNP220.6-20 | Insertion of 63 bp | AGTCCTTCGAATGGAAAGTTCTTTCATTCCGGCATGAGGGCGGC CACCGTACCCCTCAATATC | Yes |
| 8 | RNP220.6-21 | Insertion of 63 bp | TGGGAGGCTAGAGAAAGGCAGCGAAACAAAGCCAAGGCAGAA CTCCCCTGGGCTTGTGTCCCT | Yes |
| 9 | RNP220.6-22 | Insertion of 69 bp | AATACGGCGCCGATCCTGTCGGGCCCTGACAGCCCAATGACCA CGTAACGTCTCGTAACGTACTTGCGC | Yes |
| 10 | RNP220.6-23 | Insertion of 86 bp | GGCCGCAAACGGATACTTGACCCCGAAGAGGTGCACAAGGATA TAGGCTCTTTGATGGTGGACCCAATCACCACGGCCTGGGTCCG | Yes |
| 11 | RNP220.6-24 | Insertion of 100 bp | TTACGTATCATCCAAAGACATCGACTCTATCAGACCCCAAAAGA AGCTAAACGATAAGCGCTACGGACCCCACGAAAGATCATTTCC GATCGTGGACCCC | No |
| 12 | RNP220.6-25 | Insertion of 103 bp | CGTCAGCCGAGAGTCCTGGAAGAGCTCGTCGAACACCGATGCG CACAGCGTCGCTTCATGGACGAAGACGACGTGCGGGTTCCAAG GGCTTCCCACCGCCTCT | Yes |
| 13 | RNP220.6-26 | Insertion of 119 bp | GCGCCGGCATCCCCTCCAAGCCAGTGATGCAAAACCGGTCCCC CGCCCACGGCCCGCGCGCAAGCCGCTCGTGGTACTCCTCCGCGC ACTCCAGCGCAAACCACGCGCAGGAAGACCAG | Yes |
| 14 | RNP220.6-27 | Insertion of 123 bp | AAAGGTTTACATAAAACTGAAGAAGGTTTAGCTTTAATAAATTA AATAGTATCAAAAATGAATTCTAAAAGAGATTCAGATTATAAT CCCTAGCTTAAAATTTTAAACAATTATAATAAACTT | No |
| 15 | RNP220.6-28 | Insertion of 149 bp | ATTGTTTCGTTTTCTCCTCGACCTGAGCCGAAGTTCATGGTTTCT CCCCCACCTGACTCGAAGCTCACGATGGAGTTTTAACTGCAAAT GCTAAGAAAAATGCTCAGGGTTAGACCTTCTATCATTTCAGATA | Yes |

| | | | | |
|---|---|---|---|---|
| | | | CAGCTGGTTTATCTTA | |
| 16 | RNP220.6-29 | Insertion of 177 bp | ATAATTTTGATAAATTTGATTTAATTTTATTGTTACTAATTTAATTATAAAAATATAGGGGGTTACAAATTAATTATTATCCAGCTGCACTTTCCAGTACAGCTACCATGCTATGACTTTTGAGATGTTACCTTAATGAATTCACTAAATCCAATAAGTTTATCATAATACTAAAAA | No |
| 17 | RNP220.6-30 | Insertion of 207 bp | TCTCAGAGCCTGCTTCATCATCTCCGGGTCCGCAGCCGTGCCGTCGCAGCCGGAGATCGAGCGACGGGCCCATCGGCGTGAGCTCCACCCGCGGCACCCGTGTCCCCGACGCCAGCAACCGAATCGTGTAAGACCGTATGGCAATTGCTAATGGCAACCTATAGCAACCCTACACAGCAGTTTCAAGTCGGTCCCTGTCTGGTCGCT | Yes |
| 18 | RNP220.6-31 | Insertion of 280 bp | TCCAATATTGCTGCCAATAATAGGCTGTCTGTATTGTAGCTGCCCGCAGCTCGCCTGTTGTTACTCGCTCATACGCGACAGCAAAGGCCGGTCTGTATTCAATGCTCCTTTCCTGCGTTGTCCACGTCTAACGAATTGGTTTTCACCCCCTTCCGCCAACGCGCTGGAACTTTTCGCTGGGGCACTCATGACACGTCACGCGCAGCTGCGTCTGAGGTCTCTTCGATTCTCGAGTCCCGCATAGCTGGCACTTCCGTGGGCGCAAACGTTGAGGAAACCG | Yes |
| 19 | RNP220.6-32 | Substitutions of 52 bp to 24 bp | CGTTCAAGGGCCACAGGTGCACCC | No |
| 20 | RNP220.6-33 | Substitutions of 261 bp to 93 bp | TCAGGTAGGACTACCCGCTGAACTTAAGCATATCAATAAGCGGAGGAAAAGAAACTAACAAGGATTCCCCTAGTAACTGCGAGTGAAGCGGGA | No |
| 21 | RNP220.6-34 | Substitutions of 46 bp to 382 bp | GTCCTGTGCGCGAGCTCCCACGTCGCTACGTCACCATGCCTCACTGAAGGAAGCCAAATCCGGAAAGCTTGGCGTCGAGACCGAGGGAAAATCTAACCCCTGCGGGGTAATCGTTTTGGGCTCGCCGTCCTGATCGTTAATTCAGTCGTTCGACAATCCTCAGCCTCTCACCAGGGGCAATCGCTTTCACATGCTCTACCCCCCCATGCAGCACGCCAACATCGCCGGTGCAGCCCACGACAATCTCGACCTCGTCGACGCGCTGGCAAGAAGACGTCAAATTAAACTACCTATCAAAGCTCATTTGCGACAAGGAGATGGAGTTCGGGGGGGGATTAGGTGCCCCCGCTGTGGCCGGAGACGAGCGCAGGTTGGCGGTTGG | Yes |
| 22 | RNP220.6-35 | Substitutions of 5 bp to 569 bp | TAGCACGCGCGAGCCAGTCGCTTTCAAAGGCGCGCCAGGACGAGGTCGCCAACATCCTGCGGGACCATACTGACTCACGTCGTATTAAACCCAACTCGCGTAACCTTTTAATCGGCGAACAGCCGAACCCTTCCAAGCTTCTACCCCCGGAGGATAGGTCGCGCACTCCCAGCAGCGGACCTTTAGTGCCCCGACCTCCACTAACAATTATGAGATATAATGGTAATAATAGAAATTATTTGATATTAATCTTAATTTAGGATTAACTATTTCCCAATATTCTTTCTACGACATATGGTTCCAAATCTATGTTCGATTTACTTAGTAAATATTTACCTACGCCTTTTAGATAAGTTAATAATAAATTATTAACTTGGAAAGGTATCTTATTTAAATTGTTTAGAGCAGTCGAGAATATTAGTCAATGCTCGTAAGAGTGAACTAGCTAACTGAAATCATAGAATTTCTTCAAATTCATGATTGTATCGTCCGATGTCGGAGAGAGAGTGGAGTCGCACGTTTAGCGA | No |

| | | | | |
|---|---|---|---|---|
| | | | TCACGCCGGACTTGCAGTGCTGTGACCACACATCAGGCACAG | |
| 23 | RNP294-23 | Insertion of 34 bp | AGAACATTTCGGAGACCTTCGCAAGGGGCGTGGG | Yes |
| 24 | RNP294-24 | Insertion of 67 bp | ACGGGTCGAAATACGGTGAGTGTTTTTTTCTTGGTCATTTATCATGGCGCAGTGCACAGGTCGGGGT | Yes |
| 25 | RNP294-25 | Insertion of 74 bp | TCCTTTACCACCGGAACTCATCATCCATGCCTTTGAGATCGCCTCGTTGAAGGTTTCAATAGGTTGTCCATCAA | Yes |
| 26 | RNP294-26 | Insertion of 105 bp | GCATAGAAACACGATGTACAATCCCCAGACCAACGATGGCAGATGTGGCAATAACGGGTTGGCATAACGCGTGAATGACAGTATAGAATGACAGTGTCCGAGACG | Yes |
| 27 | RNP294-27 | Insertion of 113 bp | ATTTATAATGTTTTTATAAGGTTATAAAAATAATTTATTATTAATCAAAGATGATAATCCCAAATAAAGTTAACCTTTTTATTTGGTTAAAAAATTATTTTTAAAAGAAGCCA | No |
| 28 | RNP294-28 | Insertion of 156 bp | CGAGCGTTTCTCTTTTGCGTTCTTCATATCCTTCGGCGCGCTGGATGTAACGTACCTGCTCGCAACCTGCTCAAAACTCAGTACTCAGAAGTGCCTGCGGGCCCGTCAAACGCGGTGTTGAACTTGAAATCCGTCGCACGCGTGTCGCCCTGGCAA | Yes |
| 29 | RNP294-29 | Insertion of 173 bp | AGTGGTGTCAGCTCTACGACGCCGAGCCGTGGATCCTGCCGGAGAATACGACGCCGGACGTCATCGAAAAGGTGAAAGAACAATTCTTAATGGACCTGAGCTGGTTCATATATTCATGGGCCGAGATTTGGCCCGAGACATCTCGAGCTCGAGGCCCTGCTCCGCCTCCTGCG | Yes |
| 30 | RNP294-30 | Insertion of 235 bp | GTCGGGGAGCACCAAGTGGTGGGGGCGCGGGGTTGCCCACCATGGGGGCAGGTTGAGCGATGGCCCATTGGCAATCAAGAGCTAATCAGAAAAGAAGATGCACTGCGAGTAGTAGTCGTTCTGCTCCGTGCAAACTGAGCCAGACGCGCAAGTCGTGGCACCAGCTATGAAAGGAAGACAAGAAATCAGGTTAGCTTGTGACTTGGACATTGAACCCGGCGACGCCACGTTAACG | No |
| 31 | RNP294-31 | Substitutions of 106 bp to 12 bp | CGTAGACGCCTG | Yes |
| 32 | RNP294-32 | Substitutions of 52 bp to 54 bp | TATTCTCACCTATTTTATTAGATAATTCTATCTTAAATGAAAATAGATTATTTA | No |
| 33 | RNP294-33 | Substitutions of 4 bp to 85 bp | GTTGGATGGAAAGGAAACGGGGGGGAAAGAGGAAGAGTGGCCCTATGCAGGGGTATGCGAGGCCACCCTGAGTCGGACTGCTGAG | Yes |
| 34 | RNP294-34 | Substitutions of 1 bp to 88 bp | GGGGTAATATCCGCGGTAAAACCCATACAGCTGCTACTACTCATAAATCTAGTACTTTTTATAAAAAAGAAACTTAAAATACTTTCTA | No |
| 35 | RNP294-35 | Substitutions of 1 bp to 121 bp | TTAGATACTCATCAGTATATTTTAACATATATCGGAAATTAGGATCATCCTTATCACCGGAACTACCGAAGTAAGTATCAAATATCTGAGTATCTCCATTCATATAGAATGATAAGAAGAA | No |

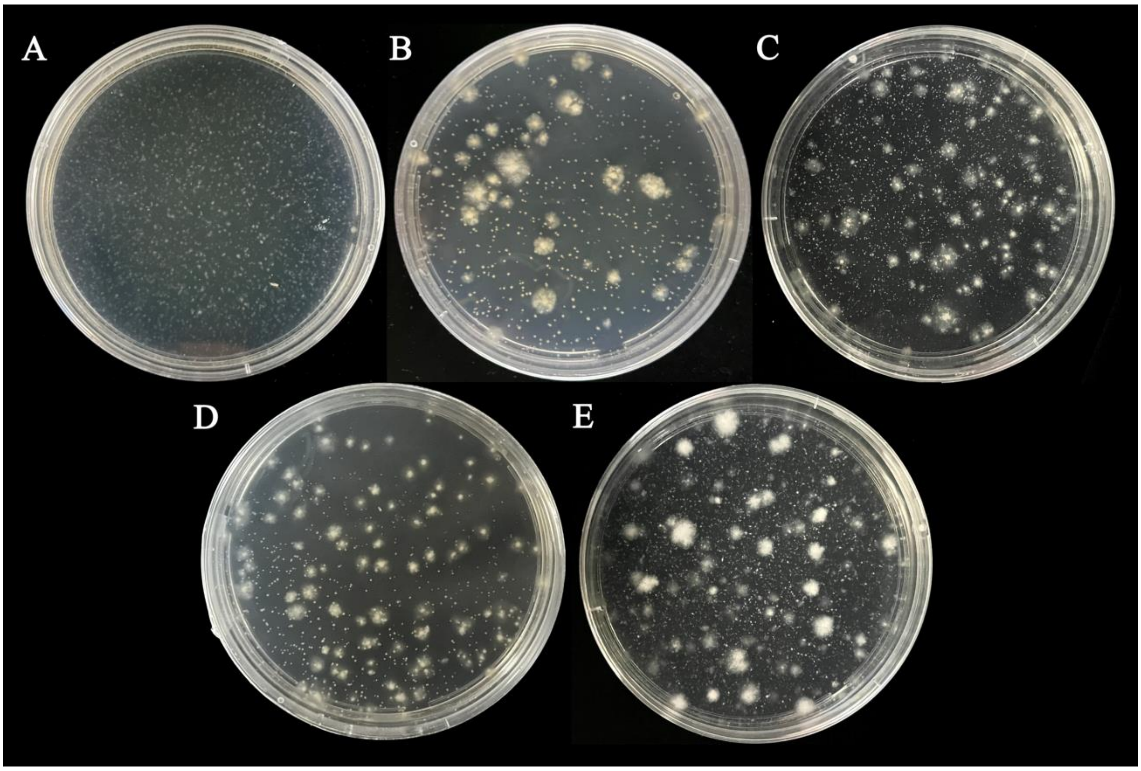


**Figure S1.** Effect of RNP concentration on the number of positive transformants. (A-E): The different concentrations of RNPs in transformation system: 0.0, 73.5, 147.0, 220.6, and 294.0 nM, respectively. The double-layered MM medium was used, with a lower medium without 5-FOA for protoplast regeneration and an upper medium containing 400 mg/L 5-FOA for the selection of edited mutants. The positive transformants would penetrate the upper layer and grow.

A

Cleavage site

WT: CAACCGGCTCATACACGGAAGAGGCCGTCCGTATGGCTCGCGCCAACCGCGACT

1: AAG--- (38 bp) ---GGCTCGCGCCAACCGCGACTTCGTGATCGGCTTCATTGCCCAGCA

2: CAACC----CATACACGGAAGAGGCCGTCCGTATGGCTCGCGCCAACCGCGACTTCGT

3: CAACCGGCT-ATACACGGAAGAGGCCGTCCGTATGGCTCGCGCCAACCGCGACTTCG

4: CAACCGGCTACATACACGGAAGAGGCCGTCCGTATGGCTCGCGCCAACCGCGACTT

5: CAACCGGCTGCATACACGGAAGAGGCCGTCCGTATGGCTCGCGCCAACCGCGACTT

6: CAACCGGCTTCATACACGGAAGAGGCCGTCCGTATGGCTCGCGCCAACCGCGACTT

7: CAACCGGCTCGCATACACGGAAGAGGCCGTCCGTATGGCTCGCGCCAACCGCGACT

8: CAACCGGCTTGT (106 bp) CCTCATACACGGAAGAGGCCGTCCGTATGGCTCGCGCCA

9: GTCTGCC (56 bp) CCGGCT→ATTAACACTACATACACGGAAGAGGCCGTCCGTATGG

10: CAACCGGCT→GAG (66 bp) CATCATACACGGAAGAGGCCGTCCGTATGGCTCGCGC

B

Cleavage site

WT: CAACCGGCTCATACACGGAAGAGGCCGTCCGTATGGCTCGCGCCAACCGCGACT

1: TTC--- (50 bp) ---GAGGCCGTCCGTATGGCTCGCGCCAACCGCGACTTCGTGATCGGC

2: CTC--- (33 bp) ---CGCGCCAACCGCGACTTCGTGATCGGCTTCATTGCCCAGCAACGC

3: CAACCGGCT-------GGAAGAGGCCGTCCGTATGGCTCGCGCCAACCGCGACTTCGTG

4: CAACCGGCT----CACGGAAGAGGCCGTCCGTATGGCTCGCGCCAACCGCGACTTCGT

5: CAACCGGCT-ATACACGGAAGAGGCCGTCCGTATGGCTCGCGCCAACCGCGACTTC

6: CAACCGGCT-ATACACGGAAGAGGCCGTCCGTATGGCTCGCGCCAACCGCGACTTC

7: CAACCGGCT-ATACACGGAAGAGGCCGTCCGTATGGCTCGCGCCAACCGCGACTTC

8: CAACCGGCTGTCATACACGGAAGAGGCCGTCCGTATGGCTCGCGCCAACCGCGAC

9: CAACCGGCTAAC (26 bp) GCGCATACACGGAAGAGGCCGTCCGTATGGCTCGCGCC

10:CAACCGGCTGAC (100 bp) ACACATACACGGAAGAGGCCGTCCGTATGGCTCGCGC

C

▼ Cleavage site

WT: CAACCGGCTCATACACGGAAGAGGCCGTCCGTATGGCTCGCGCCAACCGCGACT
1: CTG--- (153 bp) ---TCGGCTTCATTGCCCAGCAACGCATGGACAGCGTCGGTCTGCGG
2: TGC--- (150 bp) ---GGGAGGGCGAGTCCTCGCCGGACGAAGACTTCCTTATCCTTACT
3: GCT--- (124 bp) ---TCATTGCCCAGCAACGCATGGACAGCGTCGGTCTGCGGGAGGGC
4: TCT--- (86 bp) ---TGATCGGCTTCATTGCCCAGCAACGCATGGACAGCGTCGGTCTGC
5: CAG-------------CATACACGGAAGAGGCCGTCCGTATGGCTCGCGCCAACCGCGACT
6: CAACCGG-----------AAGAGGCCGTCCGTATGGCTCGCGCCAACCGCGACTTCGTGAT
7: CAACCGGCT-ATACACGGAAGAGGCCGTCCGTATGGCTCGCGCCAACCGCGACTTC
8: CAACCGGCT-ATACACGGAAGAGGCCGTCCGTATGGCTCGCGCCAACCGCGACTTC
9: CAACCGGCT-ATACACGGAAGAGGCCGTCCGTATGGCTCGCGCCAACCGCGACTTC
10: CAACCGGCT-ATACACGGAAGAGGCCGTCCGTATGGCTCGCGCCAACCGCGACTT
11: CAACCGGCT-ATACACGGAAGAGGCCGTCCGTATGGCTCGCGCCAACCGCGACTTC
12: CAACCGGCT-ATACACGGAAGAGGCCGTCCGTATGGCTCGCGCCAACCGCGACTTC
13: CAACCGGCT-ATACACGGAAGAGGCCGTCCGTATGGCTCGCGCCAACCGCGACTTC
14: CAACCGGCT-ATACACGGAAGAGGCCGTCCGTATGGCTCGCGCCAACCGCGACTTC
15: CAACCGGCTTCATACACGGAAGAGGCCGTCCGTATGGCTCGCGCCAACCGCGACT
16: CAACCGGCTCCATACACGGAAGAGGCCGTCCGTATGGCTCGCGCCAACCGCGACT
17: CAACCGGCTCCATACACGGAAGAGGCCGTCCGTATGGCTCGCGCCAACCGCGACT
18: CAACCGGCTGTCATACACGGAAGAGGCCGTCCGTATGGCTCGCGCCAACCGCGAC
19: CAACCGGCTATC (22 bp) TATCATACACGGAAGAGGCCGTCCGTATGGCTCGCGCCA
20: CAACCGGCTAGT (57 bp) ATCCATACACGGAAGAGGCCGTCCGTATGGCTCGCGCCA
21: CAACCGGCTTGG (57 bp) CCTCATACACGGAAGAGGCCGTCCGTATGGCTCGCGCCA
22: CAACCGGCTAAT (63 bp) CGCCATACACGGAAGAGGCCGTCCGTATGGCTCGCGCCA
23: CAACCGGCTGGC (80 bp)CCGCATACACGGAAGAGGCCGTCCGTATGGCTCGCGCCA
24: CAACCGGCTTTA (94 bp) CCCCATACACGGAAGAGGCCGTCCGTATGGCTCGCGCCA
25: CAACCGGCTCGT (97 bp) TCTCATACACGGAAGAGGCCGTCCGTATGGCTCGCGCCA
26: CAACCGGCTGCG (113 bp) CAGCATACACGGAAGAGGCCGTCCGTATGGCTCGCGCC
27: CAACCGGCTAAA (117 bp) CTTCATACACGGAAGAGGCCGTCCGTATGGCTCGCGCC
28: CAACCGGCTATT (143 bp) TTACATACACGGAAGAGGCCGTCCGTATGGCTCGCGCC
29: CAACCGGCTATA (171 bp) AAACATACACGGAAGAGGCCGTCCGTATGGCTCGCGCC
30: CAACCGGCTTCT (201 bp) GCTCATACACGGAAGAGGCCGTCCGTATGGCTCGCGCC
31: CAACCGGCTTCC (274 bp) CCGCATACACGGAAGAGGCCGTCCGTATGGCTCGCGCC
32: CAGGGGG (46 bp) GCT→CGT (18 bp) CCCCATACACGGAAGAGGCCGTCCGTATGGC
33: GAACGC (255 bp) AGG→TCA (87 bp) GGAAGTCGGACTGGATACTAGGGGAGATAGC
34: CTTCTG (37 bp) CCGGCT→GTC (376 bp) TGGCATACACGGAAGAGGCCGTCCGTATG
35: CAACCGGCTCATAC→TAG (563 bp) CAGACGGAAGAGGCCGTCCGTATGGCTCGCG

D

Cleavage site

WT: CAACCGGCTCATACACGGAAGAGGCCGTCCGTATGGCTCGCGCCAACCGCGACT

1: GCA--- (390 bp) ---GTCCGCGCACAGGCGGAGAGGTACCGCGCAGAAGGCTGGAAGG

2: AGG--- (160 bp) ---CAGCAACGCATGGACAGCGTCGGTCTGCGGGAGGGCGAGTCCTC

3: CCG--- (150 bp) ---GCATGGACAGCGTCGGTCTGCGGGAGGGCGAGTCCTCGCCGGAC

4: CGG--- (117 bp) ---CTTCATTGCCCAGCAACGCATGGACAGCGTCGGTCTGCGGGAGG

5: GCC--- (89 bp) ---CCAACCGCGACTTCGTGATCGGCTTCATTGCCCAGCAACGCATGGA

6: CAG--- (68 bp) ---TTGCCCAGCAACGCATGGACAGCGTCGGTCTGCGGGAGGGCGAGT

7: CGG--- (39 bp) ---CTTCGTGATCGGCTTCATTGCCCAGCAACGCATGGACAGCGTCGGT

8: CAACCGGCT-ATACACGGAAGAGGCCGTCCGTATGGCTCGCGCCAACCGCGACTTCG

9: CAACCGGCT-ATACACGGAAGAGGCCGTCCGTATGGCTCGCGCCAACCGCGACTTCG

10: CAACCGGCT-ATACACGGAAGAGGCCGTCCGTATGGCTCGCGCCAACCGCGACTTC

11: CAACCGGCT-ATACACGGAAGAGGCCGTCCGTATGGCTCGCGCCAACCGCGACTTC

12: CAACCGGCT-ATACACGGAAGAGGCCGTCCGTATGGCTCGCGCCAACCGCGACTTC

13: CAACCGGCT-ATACACGGAAGAGGCCGTCCGTATGGCTCGCGCCAACCGCGACTTC

14: CAACCGGCT-ATACACGGAAGAGGCCGTCCGTATGGCTCGCGCCAACCGCGACTTC

15: CAACCGGCT-ATACACGGAAGAGGCCGTCCGTATGGCTCGCGCCAACCGCGACTTC

16: CAACCGGCT-ATACACGGAAGAGGCCGTCCGTATGGCTCGCGCCAACCGCGACTTC

17: CAACCGGCT-ATACACGGAAGAGGCCGTCCGTATGGCTCGCGCCAACCGCGACTTC

18: CAACCGGCT-ATACACGGAAGAGGCCGTCCGTATGGCTCGCGCCAACCGCGACTTC

19: CAACCGGCT-ATACACGGAAGAGGCCGTCCGTATGGCTCGCGCCAACCGCGACTTC

20: CAACCGGCT-ATACACGGAAGAGGCCGTCCGTATGGCTCGCGCCAACCGCGACTTC

21: CAACCGGCTTCATACACGGAAGAGGCCGTCCGTATGGCTCGCGCCAACCGCGACT

22: CAACCGGCTGTCATACACGGAAGAGGCCGTCCGTATGGCTCGCGCCAACCGCGAC

23: CAACCGGCTAGA (28 bp) GGGCATACACGGAAGAGGCCGTCCGTATGGCTCGCGCC

24: CAACCGGCTACG (61 bp) GGTCATACACGGAAGAGGCCGTCCGTATGGCTCGCGCC

25: CAACCGGCTTCC (68 bp) CAACATACACGGAAGAGGCCGTCCGTATGGCTCGCGCC

26: CAACCGGCTGCA (99 bp) ACGCATACACGGAAGAGGCCGTCCGTATGGCTCGCGCC

27: CAACCGGCTATT (107 bp) CCACATACACGGAAGAGGCCGTCCGTATGGCTCGCGCC

28: CAACCGGCTCGA (150 bp) CAACATACACGGAAGAGGCCGTCCGTATGGCTCGCGCC

29: CAACCGGCTAGT (167 bp) GCGCATACACGGAAGAGGCCGTCCGTATGGCTCGCGCC

30: CAACCGGCTGTC (229 bp) ACGCATACACGGAAGAGGCCGTCCGTATGGCTCGCGCC

31: CAACCGGCTCATA (100 bp) TCC→CGTAGACGCCTGTCGCCGGACGAAGACTTCCTT

32: CAGGGG (44 bp) CCGGC→TAT (48 bp) TTATCATACACGGAAGAGGCCGTCCGTATGG

33: CAACCGGCTCATA→GTT (79 bp) GAGCACGGAAGAGGCCGTCCGTATGGCTCGCGC

34: CAACCGGCTC→GGG (82 bp) CTAATACACGGAAGAGGCCGTCCGTATGGCTCGCGC

35: CAACCGGCTC→TTA (115 bp) GAAATACACGGAAGAGGCCGTCCGTATGGCTCGCGC

**Figure S2.** Verifying *ura3* mutation after RNP-based gene editing. Sequences of *G. Lucidum ura3* mutants near the target sequence obtained by transformation with 73.5 nM RNPs (A), 147.0 nM RNPs (B), 220.6 nM RNPs (C) or 294.0 nM RNPs (D). WT: wild type strain L1. The PAM sequence was highlighted in yellow. The red triangles indicated the cleavage site. Mutation type of deletion,

insertion and substitution was indicated by red line segments, red letters and red arrows respectively. The green letters before and after the red arrow represented the sequences before and after the substitution.

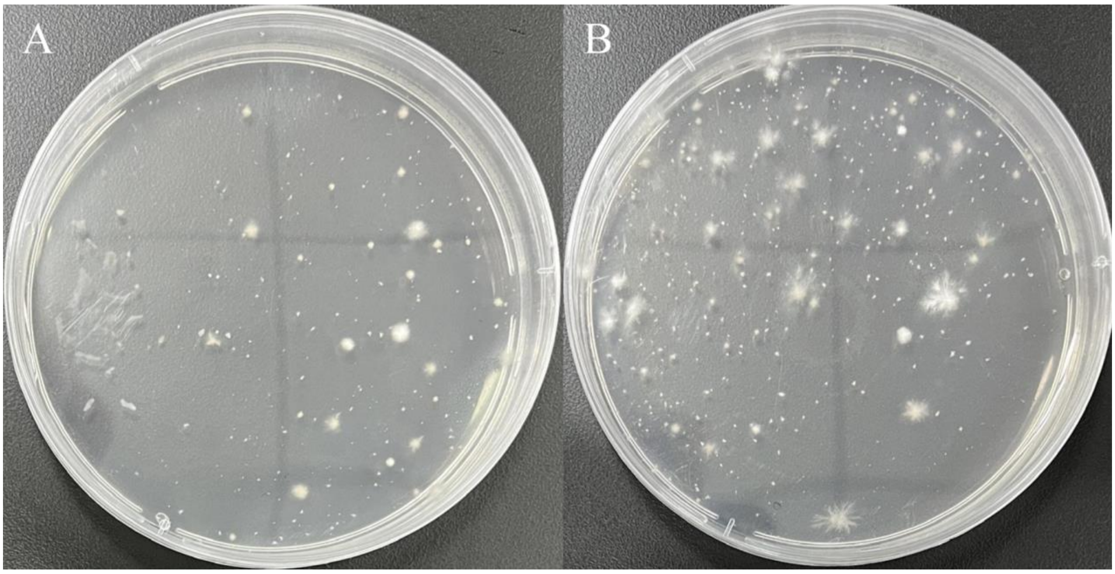


**Figure S3.** Screening of transformants with edited functional genes and added *ura3* on uracil-free MM medium. The transformants were obtained by transformation with 220.6 nM RNPs, 10 μg donor DNA and $10^7$ L1-Δ*ura3* protoplasts. A: The transformants used for screening *cyp512a3* edited mutants. B: The transformants used for screening *cyp5359n1*edited mutants.
